# Shaping attitudes toward body-related cues via evaluative conditioning: investigating the roles of social comparison and self-esteem

**DOI:** 10.1007/s10339-026-01376-y

**Published:** 2026-07-11

**Authors:** Patrycja Uram, Robert Balas

**Affiliations:** Institute of Psychology, Polish Academy of Sciences, Warsaw, Poland

**Keywords:** Evaluative conditioning, Body image, Self-esteem, Social comparison, Moderation

## Abstract

Evaluative conditioning (EC) is a mechanism through which attitudes toward neutral stimuli can be shaped by pairing them with positive or negative cues. Despite growing evidence for its role in attitude formation, little is known about whether EC can effectively modify body-related evaluations and how individual dispositions may moderate these effects. The present study examined whether neutral geometric figures (CS) would become more positive in valence and liking when paired with body images differing in weight status (average-weight vs. obese). A total of *N* = 146 participants completed baseline ratings of neutral CS, underwent an EC task pairing CS with average-weight or obese body images, and provided post-conditioning evaluations along with measures of self-esteem and body, eating and exercise-related social comparison (BEECOM). Results showed that CS paired with average-weight bodies increased significantly in both valence and liking, whereas obese-paired CS showed no significant change. Critically, social comparison tendency moderated overall responsiveness to conditioning: participants low in BEECOM showed significant increases in liking across conditions, whereas high-BEECOM participants showed no change. Neither variable moderated the specific differential conditioning effect between pairing conditions. Self-esteem did not moderate conditioning effects. Exploratory analyses revealed no evidence that conditioning altered perceptual judgments of stimulus size. Findings highlight the importance of individual differences in social comparison as determinants of conditioning responsiveness in the body image domain.

**Supplementary Information:**

The online version contains supplementary material available at https://doi.org/10.1007/s10339-026-01376-y.

## Introduction

Body image concerns represent a significant and pervasive issue in modern societies, associated with substantial psychological costs including low self-esteem, depressive symptoms, and disordered eating (Cash and Smolak 2011; Stice and Shaw 2002; Thompson et al. 1999). In contemporary digital contexts, these concerns have been exacerbated by the proliferation of social media platforms (Holland and Tiggemann 2016; Perloff 2014). These platforms dramatically increase individuals’ exposure to idealized and highly curated body images, creating unprecedented opportunities for appearance-related social comparison (Festinger 1954). Such comparisons, particularly upward comparisons to perceived ideals, activate evaluative processes that consistently predict negative affective responses and reduced body satisfaction (Fardouly et al. 2015; Tiggemann and Slater 2013; E. A. Vogel et al. 2014). Experimental evidence confirms that state appearance comparison mediates the negative impact of exposure to idealized images, with trait-level comparison tendencies moderating and often amplifying these effects (Fioravanti et al. 2022; Myers and Crowther 2009). Understanding the psychological mechanisms through which repeated exposure to body-related cues shapes evaluative responses is therefore essential for developing interventions that can effectively counteract these pervasive negative influences.

If body dissatisfaction is shaped and maintained through automatic evaluative responses formed via repeated exposure to body-related cues, then effective interventions may need to target these automatic associations directly. Evaluative conditioning (EC) represents a mechanism particularly well-suited to this goal. EC is a process through which a neutral conditioned stimulus (CS) acquires the valence of a repeatedly paired unconditioned stimulus (US; De Houwer et al. 2001; Jones et al. 2010). Critically, EC can operate through relatively automatic processes that do not require conscious deliberation (Gawronski and Walther 2012; Hütter and Sweldens 2013), paralleling the automatic nature of the evaluative responses that underlie body dissatisfaction itself. A landmark meta-analysis confirmed that EC produces robust shifts in affective responses across diverse domains including consumer preferences, political attitudes, and intergroup stereotypes (Hofmann et al. 2010). Theoretically, these effects are explained by affect-transfer models, which propose that the affective response elicited by the US becomes directly associated with the CS representation (Gawronski and Walther 2012; Jones et al. 2010; Walther et al. 2009). By leveraging the same associative learning processes through which negative body-related evaluations may form, EC offers a theoretically grounded approach for modifying these entrenched automatic responses (Martijn et al. 2010).

Given EC’s demonstrated capacity to modify affective associations, researchers reasoned that it could be applied to reshape body-related evaluations. The logic was straightforward: if body dissatisfaction reflects negative automatic associations with one’s own body, then repeatedly pairing self-representations with positive affective cues should create more positive associations via affect transfer. Early studies tested this directly by pairing participants’ own body photographs (as CSs) with positively valenced social stimuli such as smiling faces (as USs), with the expectation that positive affect would transfer from the smiles to the body representations (Aspen et al. 2015; Martijn et al. 2010). Initial findings in non-clinical samples of body-concerned women appeared promising, showing increases in explicit body satisfaction following such conditioning procedures. However, this optimism proved premature. More recent, methodologically rigorous investigations, including the first randomized controlled trial in a clinical population have failed to replicate these benefits. A multi-session online EC intervention for adolescents with eating disorders yielded no specific improvements on explicit body image measures or implicit self-associations relative to placebo control (Glashouwer et al. 2018), casting doubt on EC’s therapeutic efficacy in populations with clinically significant body image disturbance.

This initial failure in a clinical sample has been followed by a broader pattern of null findings across multiple methodologically rigorous investigations. These more recent studies addressed several limitations of early positive findings by employing larger sample sizes, pre-registered designs, placebo or active control conditions, and systematic manipulation of procedural variables. Despite these improvements in methodological rigor, the null pattern has persisted. A well-powered direct replication of the original smiling-face paradigm in undergraduate women found no advantage over placebo control (Glashouwer et al. 2019). Attempts to enhance ecological validity through a gamified mobile-app version likewise yielded no benefits beyond a neutral control group, with effects potentially attributable to mere exposure rather than conditioning per se (Kosinski 2019; Zajonc 2001). Furthermore, efforts to update the affective cues by substituting contemporary social stimuli, such as Facebook “likes” and positive emojis, for smiling faces have also failed to produce reliable valence transfer to body images or improvements in body satisfaction (Masselman et al. 2022). This pattern of null results across variations in stimulus type, delivery format, and population suggests that EC’s effectiveness in the body image domain is not simply a matter of optimizing the specific conditioned or unconditioned stimuli. Instead, the presence versus absence of effects may depend on boundary conditions that operate at a different level of analysis. Theoretically, two categories of moderators warrant systematic investigation: stimulus-level factors related to the properties or meaning of specific CS-US pairings, and person-level factors reflecting individual differences in susceptibility to or engagement with the conditioning procedure itself (De Houwer 2018; Moran et al. 2023). The latter remain almost entirely unexplored in the body image EC literature.

Social comparison was treated as the more directly relevant individual-difference variable in the present study. Appearance-related social comparison is closely tied to body-image outcomes, and a broad literature indicates that frequent upward appearance comparison is associated with greater body dissatisfaction and related concerns (Fardouly et al. 2015; Myers and Crowther 2009; Vartanian and Dey 2013). In the context of the present paradigm, this suggests that individuals higher in social comparison tendency may process body-related unconditioned stimuli (USs) in a more personally meaningful way, making social comparison a plausible moderator of evaluative change. At the same time, because prior work offers multiple plausible mechanisms and does not support a precise directional prediction for the present task, social comparison was examined as an exploratory moderator rather than as the basis for a strongly directional hypothesis.

The rationale for including self-esteem was more tentative than that for social comparison. Pairing procedures and evaluative-conditioning interventions have previously been examined in relation to self-evaluative outcomes, including self-esteem, and body-image interventions in this tradition have sometimes reported changes in self-esteem alongside changes in body satisfaction (Dijksterhuis 2004; Halbeisen and Paslakis, 2021; Martijn et al. 2010). However, this literature provides only limited guidance on whether global trait self-esteem should moderate evaluative-conditioning effects in a paradigm using neutral geometric figures as conditioned stimuli. We therefore included self-esteem as an exploratory individual-difference variable because of its broader clinical relevance to body image and because prior pairing-based interventions have implicated self-evaluative outcomes more generally, rather than because there is a strong a priori theory linking global self-esteem to associative learning in the present task. Accordingly, no directional prediction was made for self-esteem moderation.

The theoretical considerations outlined above converge on a critical gap: despite strong conceptual rationales, neither social comparison tendency nor self-esteem has been systematically examined as a moderator of EC effects in the body image domain. These two variables emerge as priority candidates for investigation because they operate through distinct and complementary mechanisms. Social comparison directly influences the processing of the body image stimuli that serve as unconditioned stimuli in EC procedures, potentially gating the affect transfer process itself. Self-esteem, by contrast, may influence the receptivity to or integration of new evaluative associations more broadly. Testing these moderators carries both theoretical and practical significance. Theoretically, moderation by social comparison would provide evidence that EC effects depend on the elaborated meaning and personal relevance attributed to the US, rather than operating purely through low-level affect transfer. Conversely, a null moderation effect would support domain-general associative learning accounts (Hofmann et al. 2010; Jones et al. 2010). Practically, identifying individual differences that predict responsiveness to EC would enable the development of personalized or stratified intervention approaches, targeting those most likely to benefit while sparing resources for alternative treatments where EC proves ineffective. Given that both social comparison and self-esteem could plausibly moderate the formation of evaluative associations, we expected these individual differences to influence changes in both valence and liking ratings of conditioned stimuli (Fardouly et al. 2015; Martijn et al. 2010). Therefore, a primary goal of the present study was to provide the first systematic test of whether social comparison tendency and self-esteem moderate the effectiveness of evaluative conditioning on affective evaluations of stimuli paired with body images.

Beyond the primary question of whether and for whom EC modifies body-related evaluations, the present study included a secondary, exploratory aim to test theoretical boundaries of the conditioning mechanism itself. While EC is traditionally conceptualized as modifying evaluative judgments such as valence and liking through affect transfer (Gawronski and Walther 2012; Hofmann et al. 2010), recent theoretical advances propose that conditioning-like procedures may also transfer specific non-evaluative attributes from the US to the CS–a process termed “attribute conditioning” (Förderer and Unkelbach 2015; Unkelbach and Förderer 2018). Emerging evidence supports this possibility: EC-like procedures have been shown to alter perceptions of non-evaluative semantic attributes such as healthiness (Quigley et al. 2025), athleticism (Högden et al. 2020), and trustworthiness (Huzoaica et al. 2025). However, this research has focused exclusively on high-level conceptual or social attributes. Whether such effects extend to basic perceptual judgments-such as perceived physical size-remains an open theoretical question with implications for understanding the scope and limits of associative learning mechanisms. The present design provided an opportunity to test this boundary: if repeatedly pairing neutral geometric figures with body images differing markedly in weight status (and thus conceptually in “size”) could alter the perceived physical dimensions of those figures, this would provide novel evidence that conceptual knowledge acquired through pairing can penetrate low-level perceptual judgments. We emphasize that this perceptual question is theoretically distinct from the body image intervention focus and serves primarily to contribute to basic conditioning theory rather than applied clinical goals.

The question of whether such attribute transfer could extend to basic perceptual judgments remains contested within cognitive science. While substantial evidence suggests that many apparent top-down influences on perception reflect post-perceptual decision biases rather than genuine perceptual changes (Firestone and Scholl 2016), some evidence indicates that conceptual knowledge can alter perceptual experience under specific conditions, particularly when conceptual associations are strong or learned through extended exposure (Lupyan 2015; Walsh et al. 2024). The present design allowed an exploratory test of this theoretical boundary: the body image stimuli provide a salient conceptual dimension of size (average-weight vs. obese bodies), raising the question of whether repeated pairing of neutral geometric figures with these categorically distinct body types might alter the perceived physical dimensions (width, height) of the figures themselves. We approached this as an open theoretical question with no directional prediction, recognizing that the weight status of photographed bodies represents a semantic/conceptual property rather than a perceptually present feature of the geometric CS.

The present study was designed to address these theoretical and empirical gaps through a systematic examination of evaluative conditioning using body image stimuli, with particular attention to individual difference moderators. We employed an EC paradigm in which neutral geometric figures (CS) were repeatedly paired with either positively-valenced average-weight body images or negatively-valenced obese-weight body images (US). We assessed changes in both evaluative (valence, liking) and perceptual (width, height) ratings from pre- to post-conditioning, and measured individual differences in self-esteem and social comparison tendency.

## Aims of the study

Based on affect-transfer models of EC (Gawronski and Walther 2012; Hofmann et al. 2010), we formulated one primary hypothesis and two exploratory research questions.

We treated the direction and magnitude of any change for CSs paired with obese bodies as an open question because the present stimulus set was not independently prevalidated prior to the study and current theory does not justify a precise a priori prediction for negative pairings in this context.

### Hypothesis 1

We predicted a significant Time × Paired Condition interaction for evaluative ratings, such that CS paired with average-weight bodies would increase in valence and liking from pre- to post-conditioning, whereas CS paired with obese bodies would either remain unchanged or decrease, reflecting the transfer of positive versus negative affect from the respective US categories. This prediction assumes bidirectional affect transfer corresponding to the valence of the US.

Research Question 1: Given the theoretical considerations but directional ambiguity outlined above, we posed an exploratory question regarding individual difference moderation: Do social comparison tendency (BEECOM) and/or self-esteem (SES) moderate the magnitude or direction of evaluative conditioning effects on valence and liking ratings? These moderation tests were treated as exploratory because theory suggests several plausible but competing mechanisms and does not support strong directional predictions.

Research Question 2: As a secondary, exploratory test of conditioning theory boundaries, we asked: Does the evaluative conditioning procedure produce corresponding changes in non-evaluative perceptual judgments of the CSs (perceived width and height)? This question concerned ratings of the CSs only and was approached as an open theoretical question without directional prediction, recognizing the contested nature of conceptual influences on perceptual judgment.

Finally, contemporary EC theory distinguishes between associative mechanisms that operate independently of awareness and propositional learning mechanisms that require conscious recognition of CS-US contingencies (De Houwer 2018; Mitchell et al. 2009). These mechanisms have different theoretical implications and may be differentially sensitive to individual differences. We therefore assessed participants’ awareness of the conditioning contingencies and any perceived influence of the body images on their ratings, enabling exploratory examination of whether observed effects depended on explicit recognition of the CS-US relationships.

## General method

The sample size was determined a priori using G*Power 3.1 (Faul et al. 2009). Because the primary hypothesis concerned the evaluative conditioning effect indexed by the Time × Paired Condition interaction in evaluative ratings, the power analysis was aligned with this pre–post within-subject contrast. Based on the body-related evaluative conditioning effect reported by Dwyer et al. (2007), we assumed an effect size of f = 0.197. Under assumptions of α = 0.05, 1 − β = 0.95, and a correlation of 0.50 among repeated measures, the required minimum sample size was *N* = 44. The final analyzed sample (*N* = 146) therefore substantially exceeded this requirement. At the same time, the study was powered primarily for the main conditioning effect and may have been less sensitive to smaller higher-order interactions or moderation effects.

To reduce respondent burden in this fully online, multi-phase protocol, the items of the BEECOM and RSES were distributed across the beginning and end of the session rather than administered in a single block. This decision was informed in part by the broader logic of experimental body-image research, in which exposure to appearance-related stimuli is often embedded between pre- and post-assessments, particularly in studies examining body-image outcomes in relation to social comparison processes (Brown and Tiggemann 2016; de Valle et al. 2021; Fardouly et al. 2015; Wilhelm et al. 2019). Importantly, BEECOM and RSES were not analyzed as pre- versus post-manipulation outcomes. Instead, all items were recombined into single full-scale scores, which were used as person-level moderators in the exploratory analyses. To further evaluate this administration format, we examined split-half reliability across the two administration halves; the resulting Spearman-Brown corrected split-half coefficients were acceptable for both scales (BEECOM = 0.86, RSES = 0.82), supporting the adequacy of the distributed administration format. At the same time, because the study was conducted as a relatively long online protocol, distributing questionnaire items across the session was also intended to reduce local respondent burden, consistent with methodological work on modular administration in self-administered surveys (West et al. 2015). Accordingly, these scores are interpreted as session-level individual-difference indicators rather than as conventional pre/post questionnaire measures.

### Data preparation

Data were collected online from 174 participants via a multi-phase Inquisit Web procedure. A total of 13 participants were excluded for failing to meet one or more of the pre-specified inclusion criteria: providing informed consent, being at least 18 years of age, completing all critical phases of the study, passing an embedded attention check, or having a duplicate entry. Subsequently, the data were screened for patterns of non-attentive responding. An additional 14 participants were excluded for exhibiting either invariant responding (i.e., a standard deviation of zero) across all items of a psychometric scale (Rosenberg Self-Esteem Scale or BEECOM) or invariant responding across all pre- and post-conditioning stimulus ratings for both valence and liking. Finally, after calculating Body Mass Index (BMI) from self-reported height and weight, one participant was removed due to a biologically implausible BMI value. This multi-stage screening procedure resulted in a final sample of 146 participants whose data were used in all subsequent analyses.

### Participants

The final analysis included 146 participants aged 18 to 52 (M = 23.90, SD = 5.90). The sample was composed of 91 women (62.3%), 52 men (35.6%), and 3 individuals (2.1%) who selected the “prefer not to say” option. The average body mass index (BMI) was 23.50 (SD = 4.19), with a range from 16.50 to 39.70. In terms of place of residence, 55 participants (37.7%) lived in cities with more than 500,000 inhabitants, 39 (26.7%) in cities with up to 100,000 inhabitants, 32 (21.9%) in rural areas, 13 (8.9%) in cities with up to 500,000 inhabitants, and 7 (4.8%) in cities with up to 250,000 inhabitants. Regarding education, 98 participants (67.1%) had completed secondary education, 42 (28.8%) had higher education, 3 (2.1%) had vocational education, 2 (1.4%) had primary education, and 1 (0.7%) had lower secondary education. Participants were recruited via online advertisements and received no compensation. The study was conducted in accordance with the Declaration of Helsinki and was approved by the local Research Ethics Committee. All participants provided informed consent prior to the experiment.

The inclusion of both women and men was intentional. First, the present design was informed in part by Dwyer et al. (2007), whose evaluative-conditioning study using body-shape stimuli also included male and female participants and reported no evidence for gender differences in the conditioning effect. Second, the broader body-image literature indicates that appearance comparison and body-image responses to body-related stimuli are relevant in both women and men. For example, Davison and McCabe (2005) examined appearance comparison and related body-image constructs in both men and women, and Rochelle and Huang (2025) used an experimental pre-exposure/post-exposure design in a mixed-gender sample to examine body satisfaction and appearance comparison following exposure to idealized body images. Accordingly, we considered the inclusion of both women and men appropriate for examining evaluative responses to body-related stimuli more generally, while acknowledging that the present design was not optimized to isolate same-gender comparison effects.

### Materials

The conditioned stimuli (CS) were eight neutral geometric figures, including circles, squares, and triangles of varying designs, taken from Gawronski et al. (2014) and presented as individual PNG image files. The unconditioned stimuli (US) were eight photographs of human bodies varying in weight status. Four depicted average-weight bodies (two male, two female), and four depicted obese-weight bodies (two male, two female). All images were standardized in size, featured a uniform background, and were of similar resolution to control for low-level visual confounds. As confirmed by the manipulation check reported in the Results section, the average-weight bodies were evaluated significantly more positively than the obese bodies, establishing their intended valence (for details, see Supplementary Materials).

### Procedure

**Fig. 1:**
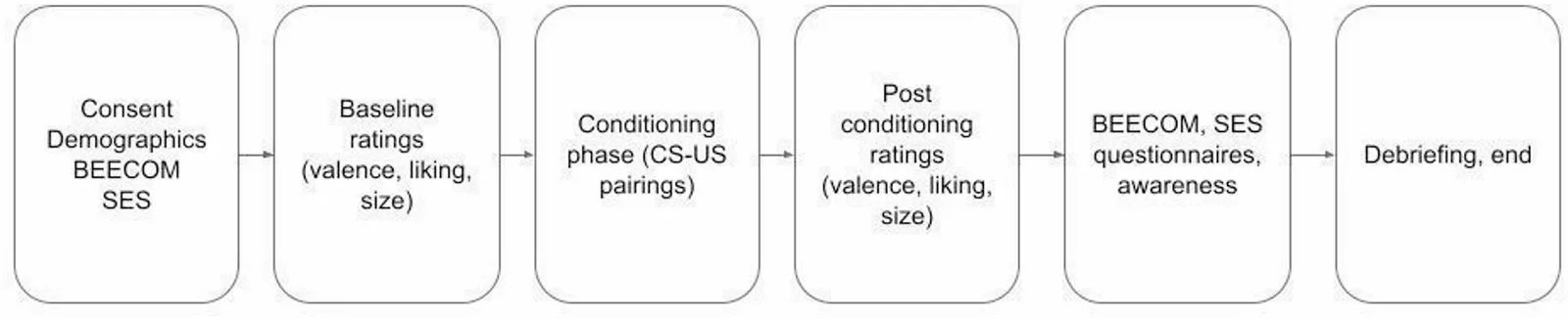
Research procedure diagram

After providing informed consent, participants engaged in a multi-phase online experiment lasting approximately 25 min (see Fig. 1).

First, participants completed a set of demographic questions, followed by the initial half of the BEECOM (6 items) and RSES (5 items) questionnaires. Next, participants entered the pre-conditioning phase, which consisted of three randomized blocks of rating tasks. In the size estimation block, each of the eight CSs was presented for 2,500 ms, after which participants estimated its width and height. In the valence and liking blocks, participants rated all eight CSs and all eight USs on the respective − 10 to + 10 scales. The order of the valence and liking blocks was counterbalanced across participants.

Participants then proceeded to the evaluative conditioning phase. In this phase, four specific CSs were consistently paired with images of obese bodies, and the other four CSs were consistently paired with images of average-weight bodies. On each of the 64 trials, a CS-US pair was presented simultaneously side-by-side for 3,750 ms, with the left/right screen position of the stimuli counterbalanced. Each unique CS-US pairing was presented eight times in a random order.

Following the conditioning phase, participants completed the post-conditioning rating tasks. They repeated the size estimation, valence rating, and liking rating tasks for all CSs in the same counterbalanced order as in the pre-conditioning phase.

Finally, participants completed the remaining halves of the BEECOM and RSES questionnaires, the three awareness check items, an embedded attention check, and additional demographic items, including self-reported height and weight. The study concluded with a debriefing procedure.

### Measures

***Evaluative ratings.*** Participants’ affective responses to all CS and US images were measured on two dimensions using on-screen sliders. Valence ratings were made on a scale ranging from − 10 (Very negative) to + 10 (Very positive). Liking ratings were made on a similar scale ranging from − 10 (I dislike it very much) to + 10 (I like it very much).

***Perceptual ratings.*** As part of a size estimation task, participants rated the perceived width and height of each CS on separate on-screen sliders ranging from 0 to 200 mm.

***Self-esteem.*** Self-esteem was measured using the 10-item Rosenberg Self-Esteem Scale (RSES; Rosenberg, 1965) in the Polish adaptation by Łaguna et al. (2007). Participants responded to items (e.g., “I feel that I have a number of good qualities”) on a 4-point scale ranging from 1 (Strongly agree) to 4 (Strongly disagree). After reverse-scoring the appropriate items, higher mean scores indicated greater self-esteem. The scale demonstrated excellent internal consistency in the present study, Cronbach’s α = 0.91.

***Social comparison tendency.*** Social comparison tendencies related to appearance, eating, and exercise were measured using the 12-item Body, Eating, and Exercise Comparison Orientation Measure (BEECOM; Fitzsimmons-Craft et al. 2012) in the Polish adaptation by Uram et al. (2026). Participants responded to items (e.g., “In social situations, I think about how my body shape compares to other people’s body shapes”) on a 7-point scale ranging from 1 (Never) to 7 (Always). Higher mean scores indicated a greater tendency to engage in social comparisons. The scale demonstrated good internal consistency, Cronbach’s α = 0.89.

***Awareness checks.*** Following the conditioning phase, participants responded to three yes/no questions to assess awareness. These included a question about general influence (“Do you think that any features of the photos had an impact on your ratings of the figures?“), a question about contingency awareness (“In your opinion, were the photos related to the figures in any way?“), and a control question about size perception (“Did you have the impression that some figures seemed larger or smaller than others for reasons unrelated to their actual size?“).

### Apparatus

The experiment was created and administered online using Inquisit 6 Web (Inquisit, 2023). Participants completed the study on their personal computers and responded using a standard mouse.

## Results

Data Analysis: All analyses were conducted in R. The primary confirmatory analyses concerned evaluative change in the conditioned stimuli (CSs). Specifically, valence and liking ratings were analyzed separately using 2 (Time: pre- vs. post-conditioning) × 2 (Paired Condition: CSs paired with average-weight vs. obese body images) mixed ANOVAs, with participant gender retained as a between-subjects factor. Baseline ratings of the unconditioned stimuli (USs) were analyzed separately as manipulation checks using mixed ANOVAs with Body Type and Stimulus Gender as within-subjects factors and Participant Gender as a between-subjects factor. Baseline ratings of the CSs were examined to verify their initial neutrality and equivalence. For baseline evaluative ratings, equivalence among the eight CS items was tested using linear mixed-effects models with CS item as a fixed effect and participant as a random intercept, followed by Holm-Bonferroni-corrected one-sample t-tests against the neutral point. Baseline perceptual equivalence of the CSs was assessed analogously using linear mixed-effects models for perceived width and height.

Moderator analyses involving BEECOM and self-esteem, awareness analyses, and all perceptual-size analyses were treated as exploratory. In the moderation models, BEECOM and self-esteem were entered as continuous variables; references to “low,” “mean,” and “high” BEECOM and RSES in the figures reflect model-based estimates at -1 SD, the sample mean, and + 1 SD rather than empirically defined participant groups. For perceptual outcomes, non-significant Time × Paired Condition effects were followed by paired-samples tests on change scores, equivalence tests (TOST), and Bayesian analyses to quantify evidence for the null hypothesis. Effect sizes are reported as generalized eta squared (ges) for ANOVA effects and Cohen’s *d* for *t*-tests. Alpha was set at 0.05 for all analyses.

Models for liking ratings yielded a singular fit, with the variance of the CS item random intercept estimating to zero. Fixed effects estimates are reported as the primary inferential basis; results were substantively identical when the random intercept for CS item was omitted.

***Manipulation check.*** Baseline ratings of the unconditioned stimuli confirmed the intended manipulation. Average-weight bodies were rated significantly more positively (valence: M = 4.22, SD = 3.12; liking: M = 4.01, SD = 3.24) than obese bodies (valence: M = − 4.08, SD = 4.11; liking: M = − 4.37, SD = 3.95), t(142) = 18.40, *p* < .001 and t(142) = 18.70, *p* < .001, respectively, and all four stimulus categories differed significantly from the neutral point of 0 in the expected directions (all Bonferroni-corrected ps < 0.001). Both analyses also yielded significant three-way Body Type × Stimulus Gender × Participant Gender interactions, indicating that evaluations were further structured by participant and stimulus gender. Full statistics and figures are reported in the Supplementary Materials.

***Baseline CS ratings.*** Baseline ratings confirmed that all eight CSs were functionally neutral and approximately equivalent prior to conditioning. Although a small omnibus difference among items reached significance for valence, F(7, 991.62) = 2.63, *p* = .011, η²ₚ = 0.018, 90% CI [0.002, 0.027], no individual CS differed from the neutral midpoint after Holm-Bonferroni correction (all padj > 0.12). For liking, neither the omnibus test, F(7, 997.82) = 1.34, *p* = .228, η²ₚ = 0.009, 90% CI [0.000, 0.014], nor any individual comparison was significant (all padj > 0.99). Full item-level statistics and figures are reported in the Supplementary Materials.

**Fig. 2:**
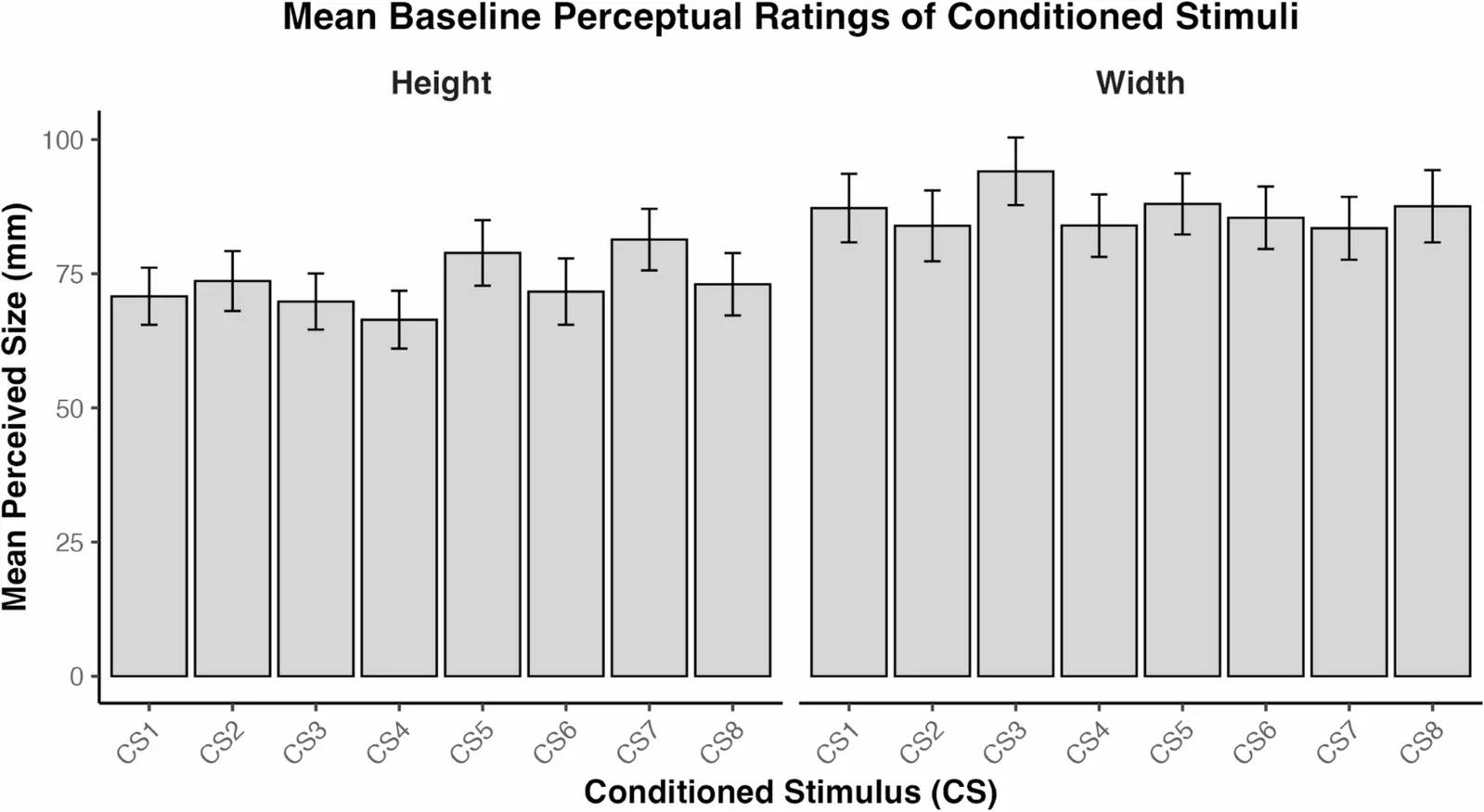
Mean baseline perceptual ratings for each conditioned stimulus (CS). The bars represent the mean perceived size (in millimeters) for each of the eight conditioned stimuli (CSs) at baseline. The left panel displays perceived height, and the right panel displays perceived width. Error bars represent 95% confidence intervals

***Baseline perceptual ratings.*** To test whether the eight conditioned stimuli (CS) were perceptually equivalent prior to conditioning, we analyzed participants’ baseline size estimations. Two separate linear mixed-effects models were conducted with either perceived width or perceived height (in millimeters) as the dependent variable, CS item as a fixed effect, and a random intercept for participant. The omnibus ANOVA revealed significant differences in perceived width among the eight CSs, F(7, 1015) = 3.54, *p* = .001, η²ₚ = 0.024, 90% CI [0.006, 0.035]. Similarly, highly significant differences were found for perceived height, F(7, 1015) = 8.45, *p* < .001, η²ₚ = 0.055, 90% CI [0.029, 0.073]. These results indicate that the stimuli were not perceptually uniform at baseline, a factor that must be considered when interpreting any post-conditioning changes in size perception. Descriptive statistics for each stimulus are presented in Fig. 2.

***Conditioning effects.*** To test the hypothesis that evaluative conditioning would alter perceptions of the conditioned stimuli (CS), we conducted two separate 2 (Time: Pre, Post) × 2 (Paired Condition: Average, Obese) × 2 (Participant Gender: Male, Female) mixed ANOVAs on participants’ aggregated valence and liking ratings. Time and Paired Condition were within-subjects factors, and Participant Gender was a between-subjects factor. The key test of the EC hypothesis is the Time × Paired Condition interaction.

**Fig. 3:**
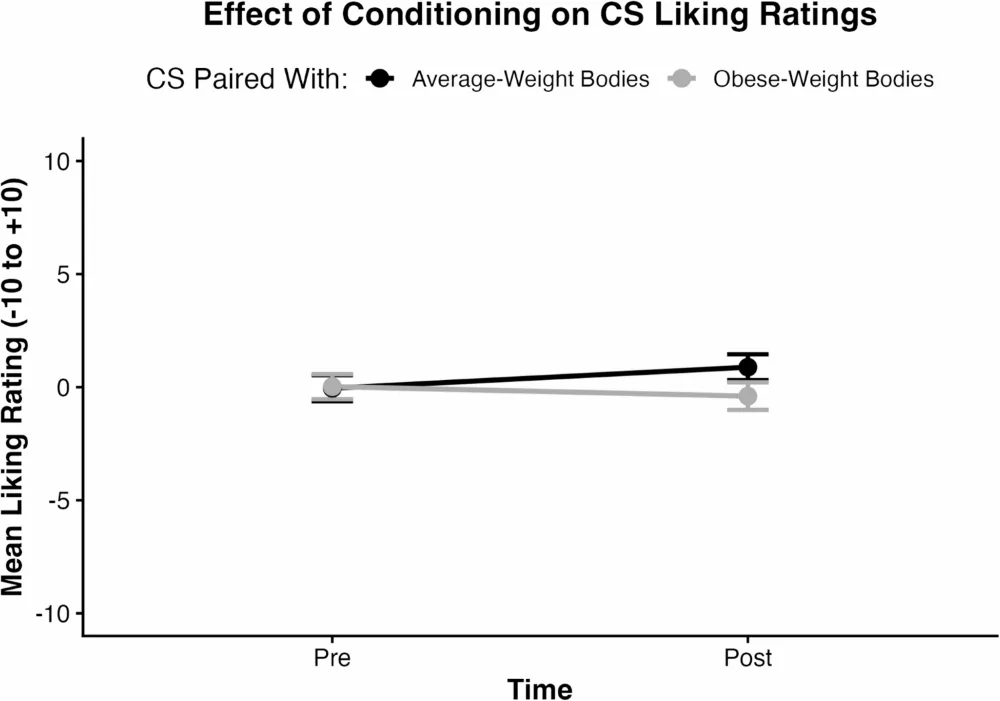
Mean liking ratings before and after conditioning as a function of paired stimulus condition. Mean liking ratings for conditioned stimuli (CSs) are shown at pre- and post-conditioning for the two experimental groups: CSs paired with average-weight bodies (black line) and CSs paired with obese-weight bodies (grey line). Error bars represent 95% confidence intervals

The analysis of liking ratings yielded a parallel pattern. The ANOVA yielded a significant Time × Paired Condition interaction, F(1, 141) = 17.17, *p* < .001, ges = 0.009, and a significant main effect of Paired Condition, F(1, 141) = 7.78, *p* = .006, ges = 0.006. No other effects were significant (all ps > 0.23). Bonferroni-corrected post-hoc comparisons to break down the interaction showed that liking ratings for CSs paired with average-weight bodies increased significantly from pre-conditioning (M = -0.05, SD = 3.48) to post-conditioning (M = 0.88, SD = 3.45), t(142) = -3.43, padj = 0.002. Liking ratings for CSs paired with obese bodies did not change significantly from pre-conditioning (M = 0.02, SD = 3.35) to post-conditioning (M = -0.40, SD = 3.69), t(142) = 1.34, padj = 0.364. This result demonstrates that the conditioning procedure also successfully increased how much participants liked the stimuli paired with average-weight bodies (see Fig. 3).

**Fig. 4:**
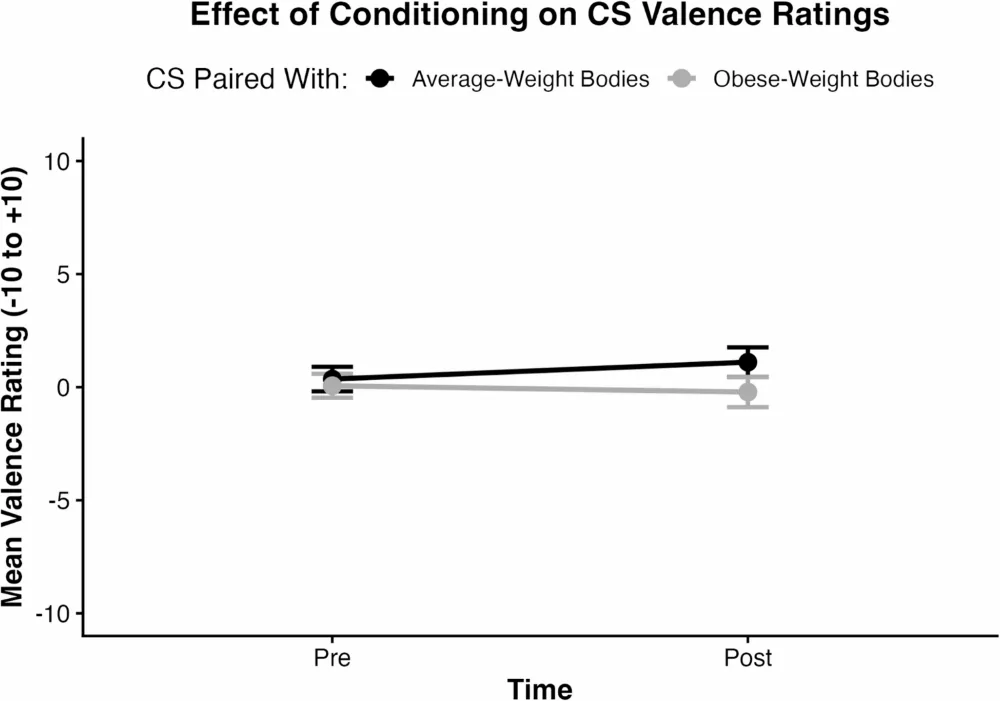
Mean valence ratings before and after conditioning as a function of paired stimulus condition. Mean valence ratings for conditioned stimuli (CSs) are shown at pre- and post-conditioning for the two experimental groups: CSs paired with average-weight bodies (black line) and CSs paired with obese-weight bodies (grey line). Error bars represent 95% confidence intervals

The analysis of valence ratings revealed a significant Time × Paired Condition interaction, F(1, 141) = 15.65, *p* < .001, ges = 0.005. There was also a significant main effect of Paired Condition, F(1, 141) = 10.23, *p* = .002, ges = 0.009. No other effects were statistically significant (all ps > 0.10). To decompose the significant interaction, we conducted Bonferroni-corrected paired-samples t-tests. As hypothesized, valence ratings for CSs paired with average-weight bodies increased significantly from pre-conditioning (M = 0.36, SD = 3.29) to post-conditioning (M = 1.10, SD = 3.96), t(142) = -2.66, padj = 0.017. In contrast, valence ratings for CSs paired with obese bodies showed no significant change from pre-conditioning (M = 0.06, SD = 3.20) to post-conditioning (M = -0.22, SD = 4.04), t(142) = 1.04, padj = 0.596. This pattern confirms that the evaluative conditioning procedure successfully increased the positive valence of neutral stimuli paired with average-weight bodies (see Fig. 4).

To address whether the gender of the body depicted in the unconditioned stimulus influenced conditioning, we extended the primary analyses by including US Gender (male vs. female body) as an additional within-subjects factor, yielding a 2 (Time) × 2 (Paired Condition) × 2 (US Gender) × 2 (Participant Gender) mixed ANOVA. For valence ratings, the Time × Paired Condition interaction remained significant, F(1, 141) = 15.82, *p* < .001, ηG² = 0.004, and no interactions involving US Gender reached significance (all ps > 0.30), including the four-way interaction, F(1, 141) = 0.03, *p* = .871. This indicates that the conditioning effect on valence operated comparably regardless of US gender or participant gender.

For liking ratings, the Time × Paired Condition interaction likewise remained significant, F(1, 141) = 18.13, *p* < .001, ηG² = 0.007. However, the four-way Participant Gender × Time × Paired Condition × US Gender interaction also reached significance, F(1, 141) = 6.20, *p* = .014, ηG² = 0.002. To decompose this interaction, we examined the three-way Time × Paired Condition × US Gender interaction separately by participant gender. Among female participants, this three-way interaction was significant, F(1, 90) = 8.77, *p* = .004, ηG² = 0.004: Holm-corrected simple effects revealed that the increase in liking for average-paired CSs was driven by stimuli paired with female bodies (Pre M = − 0.10, Post M = 1.71, *p* < .001), whereas CSs paired with male average-weight bodies showed no significant change (*p* = .475). Among male participants, the three-way interaction was not significant, F(1, 51) = 0.75, *p* = .391, indicating that the conditioning effect on liking did not differ by US gender for men, although the overall Time × Paired Condition interaction remained significant, F(1, 51) = 6.57, *p* = .013, ηG² = 0.011. This pattern suggests that same-gender body stimuli were more effective as unconditioned stimuli for liking ratings among female participants, consistent with evidence that appearance comparisons are primarily directed toward same-gender targets.

***Conditioning effects on size perceptions***. To test the hypothesis that evaluative conditioning would alter the perceived size of the conditioned stimuli (CS), we conducted two 2 (Time: Pre, Post) × 2 (Paired Condition: Average, Obese) repeated-measures ANOVAs. The dependent variables were participants’ mean ratings of perceived width and perceived height, respectively.

The analysis of perceived width revealed no significant effects. Crucially, the Time × Paired Condition interaction was not significant, F(1, 145) = 1.62, *p* = .206, ges = 0.0004, indicating that the change in perceived width from pre- to post-conditioning did not differ between CSs paired with average bodies and those paired with obese bodies. There were also no significant main effects of Time, F(1, 145) = 0.47, *p* = .492, ges = 0.0003, or Paired Condition, F(1, 145) = 0.94, *p* = .335, ges = 0.0002. These results suggest that the evaluative conditioning procedure had no influence on participants’ perception of stimulus width.

The analysis of perceived height also showed no significant Time × Paired Condition interaction, F(1, 145) = 0.71, *p* = .401, ges = 0.0001. This indicates that, as with width, the conditioning manipulation did not differentially affect the perception of height for the two CS groups. However, the analysis did yield significant main effects. There was a significant main effect of Time, F(1, 145) = 5.00, *p* = .027, ges = 0.003, indicating that, overall, stimuli were perceived as taller at post-conditioning (M = 76.50, SD = 32.40) than at pre-conditioning (M = 73.20, SD = 30.15). There was also a significant main effect of Paired Condition, F(1, 145) = 5.97, *p* = .016, ges = 0.001, which reflects the pre-existing baseline difference in the stimulus sets, with CSs assigned to the Obese condition being perceived as taller on average than those assigned to the Average condition. Given the non-significant interaction, these main effects suggest that while a general re-testing effect may have occurred for height perception, it was not specific to the conditioning manipulation.

To further probe the null results, we conducted paired-samples t-tests on change scores, formal equivalence tests (TOST), and Bayesian analyses. These converging analyses consistently supported the absence of a meaningful conditioning effect on size perception. Full details are reported in the Supplementary Materials.

In summary, despite some general changes in height perception over the course of the experiment, the core hypothesis was not supported: evaluative conditioning did not selectively alter participants’ perceptual judgments of the size of the conditioned stimuli.

### Individual differences in EC effects and size perception

To systematically investigate the role of self-esteem (SES) and social comparison tendencies (BEECOM) as potential moderators, we conducted a series of analyses to test their influence on baseline ratings, the evaluative conditioning effect, and perceptual judgments.

First, we examined the zero-order correlations between the individual difference measures and the mean baseline ratings of the stimuli. Self-esteem and social comparison were moderately and negatively correlated, r(144) = − 0.30, *p* < .001. Neither SES nor BEECOM was significantly correlated with baseline liking or valence ratings of the unconditioned stimuli (US). For the conditioned stimuli (CS), the correlation between BEECOM and baseline valence ratings did not survive Holm-Bonferroni correction for multiple comparisons and is therefore not interpreted further.

**Fig. 5:**
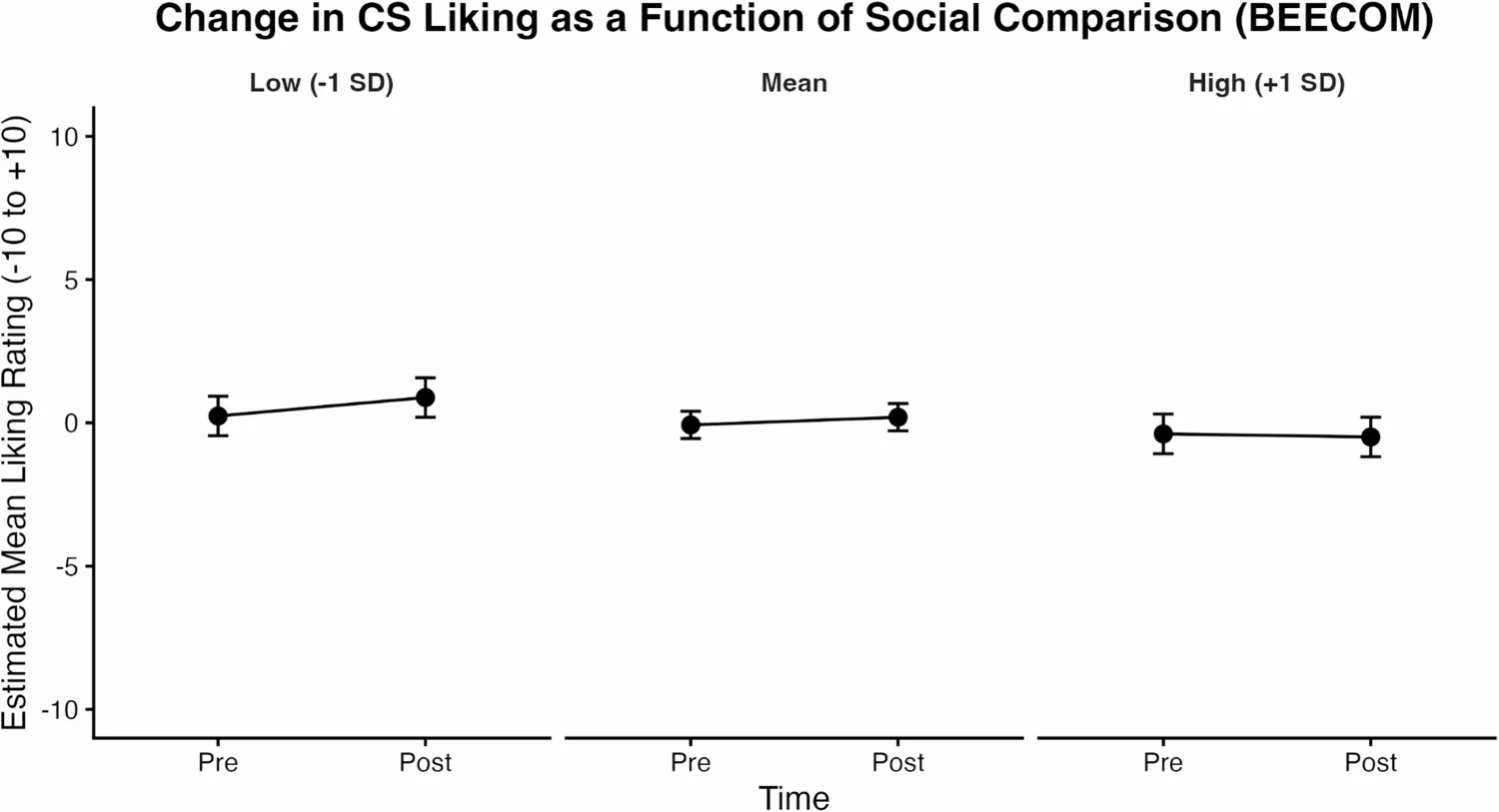
Change in mean liking ratings from pre- to post-conditioning at low, mean, and high levels of social comparison tendency (BEECOM). The figure displays the estimated marginal means for CS liking ratings before and after conditioning, separately for participants with low (-1 SD), mean, and high (+ 1 SD) scores on the BEECOM scale. Error bars represent 95% confidence intervals

To test whether individual differences moderated the evaluative conditioning effect, we fit two linear mixed-effects models, one for valence and one for liking ratings of the CSs. Each model included the full factorial structure of Time × Paired Condition × (SES + BEECOM). For valence, none of the crucial three-way interactions involving the moderators were significant (all ps > 0.19). This indicates that neither self-esteem nor social comparison tendency significantly moderated the specific evaluative conditioning effect on valence ratings. For liking, the three-way interactions were also not significant (ps > 0.08). However, the analysis revealed an unexpected but significant two-way interaction between Time × BEECOM, F(1, 2163.24) = 5.38, *p* = .020. This suggests that the general change in liking from pre- to post-conditioning (collapsing across the Average and Obese conditions) depended on participants’ social comparison tendencies. Simple slopes analysis (see Fig. 5) showed that participants with low social comparison tendencies (-1 SD) showed a significant increase in liking from pre- to post-conditioning (est. = -0.65, *p* < .001). This effect was marginal for participants with average BEECOM scores (est. = -0.27, *p* = .08) and was not present for participants with high social comparison tendencies (+ 1 SD; est. = 0.11, *p* = .63).

To address whether this pattern reflected differential general reactivity to the body stimuli rather than a specific moderation of the conditioning process, we re-estimated the liking model with each participant’s mean baseline US liking rating entered as an additional covariate. BEECOM was not significantly correlated with baseline US evaluations (*r* = − .06, *p* = .466 for liking; *r* = − .10, *p* = .248 for valence), indicating that social comparison tendency was unrelated to how participants initially evaluated the body images. Critically, the Time × BEECOM interaction remained essentially unchanged when controlling for baseline US liking, F(1, 2163.16) = 5.38, *p* = .021, demonstrating that BEECOM’s moderation of overall conditioning responsiveness was independent of participants’ general evaluative reactions to the unconditioned stimuli.

This pattern reveals that social comparison tendency moderated general responsiveness to the conditioning procedure rather than determining which specific stimulus pairings produced stronger effects. Participants low in social comparison exhibited reliable conditioning effects across both pairing conditions, whereas participants high in social comparison showed resistance to conditioning regardless of whether stimuli were paired with average-weight or obese bodies. This finding suggests that trait social comparison may influence the fundamental capacity for affect transfer in the context of body-related stimuli, rather than selectively gating responses to particular US valences. The absence of three-way interactions confirms that BEECOM did not moderate the relative effectiveness of average-weight versus obese pairings, but instead affected whether conditioning occurred at all.

Because the moderators were person-level variables, we additionally tested the moderator × gender question at the participant level by regressing each participant’s pre-to-post change in CS evaluation on the moderator, participant gender, and their interaction. We examined both overall, condition-general responsiveness and the differential conditioning score, defined as average-paired minus obese-paired change. The three participants who selected “prefer not to say” were omitted from these analyses, resulting in an analyzed sample of *n* = 143. Item-level linear mixed models including participant gender yielded the same substantive conclusions and are reported in the analysis script.

These analyses revealed a single gender-dependent effect. The previously reported moderation of overall liking responsiveness by social comparison was concentrated among women: the BEECOM × Gender interaction on overall change in liking was significant, b = 3.04, t(139) = 3.11, *p* = .002, Holm-adjusted *p* = .018 across the family of eight interaction tests. Among women (*n* = 91), higher BEECOM was associated with smaller increases in liking, simple slope = − 2.09, *r* = − .28, *p* = .007, consistent with the overall pattern described above. Among men (*n* = 52), the association was non-significant and descriptively reversed in sign, simple slope = 0.96, *r* = .25, *p* = .074.

Critically, participant gender did not qualify the differential conditioning effect, BEECOM × gender on the differential score: t(139) = − 0.20, *p* = .84. Participant gender also did not interact with self-esteem on any outcome, all ps ≥ 0.27, and the BEECOM × Gender effect for valence was not significant, t(139) = 1.59, *p* = .11. Participant gender also had no main effect on the conditioning effect itself, whether overall or differential, all ps ≥ 0.26. Thus, including participant gender did not alter the central conclusions: social comparison continued to moderate general responsiveness rather than the differential conditioning effect, whereas self-esteem remained a non-moderator.

Finally, we tested whether individual differences moderated the (null) effect of conditioning on size perception. For perceived width, there was a significant two-way interaction between Paired Condition × SES, F(1, 2175.00) = 4.21, *p* = .040. For perceived height, the same Paired Condition × SES interaction was also significant, F(1, 2175.00) = 4.16, *p* = .041. No other effects involving the moderators were significant. These interactions suggest that self-esteem was related to the pre-existing baseline differences in how the two sets of stimuli were perceived. The significant F-tests indicate a linear trend: as self-esteem increases, the perceptual difference between the two CS sets changes. However, simple slopes analyses at discrete levels of SES (low, mean, and high) did not reveal significant differences in perceived width or height between the “Average” and “Obese” CS sets at any specific level of SES. Crucially, the non-significant three-way interactions confirm that neither SES nor BEECOM moderated any change in size perception over time.

**Awareness.** To provide a robust test of whether awareness moderated the conditioning effects, we conducted a series of linear mixed-effects models. For the evaluative outcomes (valence and liking), we fit a single model for each dependent variable to test if the EC effect (the Time × Paired Condition interaction) was moderated by either general influence awareness (Q1) or contingency awareness (Q2), while statistically controlling for the other. For the perceptual outcomes (width and height), we tested if the null effect was moderated by responses to the control question about size perception (Q3). All models included random intercepts for participants and CS items.

For valence ratings, the crucial three-way interactions were not significant for either the Time × Paired Condition × Q1 interaction, F(1, 2151.07) = 0.20, *p* = .656, or the Time × Paired Condition × Q2 interaction, F(1, 2151.04) = 0.75, *p* = .387. Similarly, for liking ratings, neither the three-way interaction with Q1, F(1, 2163.31) = 2.98, *p* = .085, nor the three-way interaction with Q2, F(1, 2163.36) = 2.19, *p* = .139, reached statistical significance. While there was a statistical trend suggesting that the Time × Paired Condition × Q1 Interaction for liking was approaching significance, the overall pattern of results from this unified analysis indicates no definitive evidence that either general influence awareness or contingency awareness significantly moderated the evaluative conditioning effect for valence or liking.

We next tested whether participants who reported an impression that the figures changed in size (Q3) showed a different pattern of perceptual ratings. For perceived width, the three-way Time × Paired Condition × Q3 interaction was not significant, F(1, 2178.00) = 0.04, *p* = .839. Likewise, for perceived height, the three-way interaction was also not significant, F(1, 2178.00) = 0.12, *p* = .729. These null results confirm that responses to the control question did not moderate the (null) effect of the conditioning procedure on size perception. Overall, the analyses provide no evidence that self-reported awareness, as measured in this study, was a significant boundary condition for the observed effects.

## Discussion

The present study examined whether evaluative conditioning could modify evaluations of neutral geometric figures paired with body images differing in weight status, and whether social comparison tendency and self-esteem would moderate these effects. Conditioning successfully altered evaluations only for stimuli paired with average-weight bodies. Obese-paired stimuli showed no significant evaluative change. Critically, social comparison tendency moderated overall responsiveness to conditioning rather than the specific differential effect between conditions. Self-esteem showed no moderation. Exploratory analyses revealed no evidence that conditioning altered perceptual judgments of stimulus size. These findings provide insights into boundary conditions of evaluative conditioning in the body image domain while raising questions about conditioning asymmetry and the role of individual differences in associative learning (De Houwer 2018; Gawronski et al. 2014; Martijn et al. 2010; Moran et al. 2023).

The asymmetric conditioning pattern warrants consideration. Pairing with average-weight bodies produced reliable increases in valence (0.36 to 1.10) and liking (-0.05 to 0.88), whereas obese-body pairings yielded only non-significant directional decreases (valence: 0.06 to -0.22; liking: 0.02 to -0.40). This asymmetry challenges bidirectional affect-transfer predictions (Hofmann et al. 2010). The manipulation check confirmed both stimulus categories possessed strong opposite valences (average-weight + 4, obese − 4), yet only positive stimuli produced reliable conditioning. Three explanations merit consideration. First, conditioned stimuli rated near neutral at baseline may have exhibited floor effects, constraining detectable negative shifts given current sample size. Second, evaluative conditioning research has documented valence asymmetries (Unkelbach et al. 2020), with positive conditioning sometimes proving more robust, though underlying mechanisms remain debated (Sperlich and Unkelbach 2024). Third, obese body images may evoke complex responses beyond simple negative valence, including disgust, moral evaluation, or cognitive conflict regarding weight stigma (Lieberman et al. 2012; Pearl et al. 2020; Vartanian 2010), disrupting straightforward affect transfer. Future research with larger samples could determine whether negative conditioning genuinely fails or current power was inadequate.

The gender-specific pattern in liking ratings provides an additional nuance. Among female participants, the conditioning effect was driven by CSs paired with same-gender (female) average-weight bodies, whereas pairings with male bodies produced no significant change. This pattern aligns with social comparison theory, which posits that appearance-related comparisons are preferentially directed toward same-gender targets (Myers and Crowther 2009). That this effect appeared for liking but not valence may reflect the greater evaluative engagement that liking judgments require relative to simple valence appraisals. However, we note that the four-way interaction, although statistically significant, accounted for a very small proportion of variance (ηG² = 0.002), and that the cell sizes, particularly for male participants (*n* = 52), limited power to detect symmetric effects. This finding should therefore be treated as preliminary and interpreted with appropriate caution.

We interpret the gender-specific BEECOM effect cautiously and explicitly as exploratory. The finding is broadly consistent with the same-gender appearance-comparison account invoked above, because appearance-related comparison processes may be more strongly engaged when body-related stimuli are personally relevant to participants. However, the male subsample was modest (*n* = 52), the within-male trend was non-significant and opposite in direction, and participant gender did not qualify the differential conditioning effect. We therefore frame this result as a hypothesis for future confirmatory, gender-balanced replication rather than as an established gender-specific moderator. The full results, including the table of all interaction tests and simple slopes, are reported in the Supplementary Materials.

The BEECOM finding also requires a restrained interpretation. Appearance-related social comparison is robustly linked to body dissatisfaction and related body-image outcomes (Fardouly et al. 2015; Myers and Crowther 2009; Vartanian and Dey 2013), which made it a plausible moderator candidate in the present study. However, social comparison did not moderate the critical Time × Paired Condition interaction. Instead, higher BEECOM scores were associated with smaller overall pre- to post-change in liking across both pairing conditions. Because this effect collapsed across condition, it is better interpreted as a difference in general responsiveness to the task or repeated exposure than as evidence for a body-image-specific moderation of evaluative conditioning. Importantly, this interpretation is not explained by individual differences in how participants evaluated the body stimuli themselves: BEECOM was uncorrelated with baseline US evaluations (rs < 0.10), and the moderation effect survived controlling for each participant’s mean baseline US liking rating. The effect therefore reflects a genuine difference in evaluative plasticity during conditioning rather than a pre-existing difference in US reactivity (Hofmann et al. 2010; Martijn et al. 2010).

One possibility is that individuals higher in appearance-related social comparison engaged more strongly in appearance-focused processing during the task, which may have dampened overall evaluative change. At the same time, the present data do not discriminate cleanly among attentional, defensive, and demand-related explanations. More process-focused measures would be needed before stronger mechanistic conclusions can be drawn (Fardouly et al. 2015; Vartanian and Dey 2013).

Evidence for self-esteem as a moderator was weaker. Self-esteem did not interact with the conditioning contrast, and any associations with baseline perceptual ratings should not be interpreted as moderation of learning. In retrospect, the rationale for self-esteem was broader and less specific than the rationale for social comparison. Pairing-based and evaluative-conditioning interventions have previously been examined in relation to self-evaluative outcomes, including self-esteem, but primarily as outcomes rather than as moderators of learning (Dijksterhuis 2004; Halbeisen and Paslakis, 2021; Martijn et al. 2010). This literature therefore provides only limited grounds for expecting global trait self-esteem to moderate evaluative-conditioning effects in the present paradigm, especially when the conditioned stimuli were neutral geometric figures rather than directly self-relevant stimuli. The present null result therefore argues against strong claims about self-esteem in this paradigm and suggests that future work should prioritize constructs more directly tied to body-related cue processing.

Exploratory investigation of perceptual effects yielded clear null findings. Evaluative conditioning did not alter perceived size, evidenced by non-significant Time × Paired Condition interactions, successful equivalence tests for height, and Bayes factors supporting the null hypothesis. An important constraint stems from baseline analyses showing conditioned stimuli were not perceptually equivalent prior to conditioning. Significant omnibus tests revealed pre-existing differences in width and height. This baseline heterogeneity with null conditioning effects provides strong evidence that pairing with different body types did not induce perceptual changes. These findings contribute to understanding attribute conditioning boundaries. While prior research documented that conditioning-like procedures transfer semantic attributes such as healthiness, athleticism, and trustworthiness (Förderer and Unkelbach 2015; Unkelbach and Förderer 2018), present results suggest effects may not extend to basic perceptual dimensions. This aligns with cognitive penetrability debates supporting limited top-down influences on low-level perceptual processing (Firestone and Scholl 2016; Pylyshyn 1999; Raftopoulos 2017).

Awareness analyses revealed no significant moderation of conditioning effects. Three-way interactions involving either general influence awareness or contingency awareness failed to reach significance for both valence and liking outcomes. This pattern is consistent with associative learning accounts emphasizing automatic affect transfer that does not require conscious recognition of contingencies. However, this interpretation must remain tentative given that awareness was assessed through crude yes/no questions rather than sensitive continuous measures or recognition tests. More sophisticated assessment methods might reveal awareness effects not detected by the present measures. The absence of awareness moderation aligns with the interpretation that conditioning in this paradigm operates through relatively automatic processes, as emphasized in the theoretical framework presented in the introduction (Gawronski and Walther 2012; Hütter and Sweldens 2013).

Integration with prior literature requires acknowledging consistencies and contradictions. The present study demonstrated evaluative conditioning effects using body-related stimuli, whereas recent rigorous investigations often yielded null findings. Several factors may account for this discrepancy. Most critically, using geometric figures rather than participants’ own body photographs represents fundamental design differences (Martijn et al. 2010; Masselman et al. 2022). Lower self-relevance of geometric stimuli may have reduced defensive processing, demand characteristics, or reactance interfering with conditioning when personally meaningful stimuli are employed. However, this design choice limits applied relevance. Demonstrating neutral shapes acquire positive associations through pairing with positively-valenced body images does not directly inform whether conditioning can improve attitudes toward one’s own body. Present findings should be interpreted as illuminating affect-transfer mechanisms and boundary conditions in evaluative conditioning theory rather than evidence for clinical efficacy of body image interventions (Glashouwer et al. 2018; Hofmann et al. 2010).

A second factor potentially explaining divergent findings involves unmeasured individual differences. Prior null-effect studies did not assess social comparison tendency, which present findings suggest moderates conditioning responsiveness. If samples differ in mean social comparison levels or proportion of high versus low comparers, this could contribute to inconsistent results. Future research should systematically measure social comparison and control for it statistically or examine it as moderator. Early positive findings employed non-clinical samples of body-concerned women, whereas the most definitive null finding came from adolescents with eating disorders. Clinical populations may differ systematically in social comparison tendency, defensive information processing, or resistance to evaluative change, any of which could moderate conditioning effectiveness (Ralph-Nearman et al. 2019; Shafran et al. 2008).

## Limitations and future research directions

Several limitations constrain interpretation and generalizability. Most fundamentally, using geometric figures rather than body representations limits direct application to body image concerns. While this enabled clean testing of affect-transfer mechanisms without self-relevance confounds, it sacrifices ecological validity (Martijn et al. 2010; Masselman et al. 2022). Whether social comparison moderation would replicate when conditioning targets participants’ own body images remains empirical. The asymmetric conditioning pattern requires explanation beyond available data. Whether obese-body pairings failed due to floor effects, inadequate power, or genuine mechanistic differences in positive versus negative affect transfer cannot be determined. Single-session laboratory design provides no information about effect persistence, critical for intervention applications. Non-clinical samples limit generalizability to populations with clinically significant body image disturbance. Finally, crude awareness measures preclude strong conclusions about contingency awareness role in mediating effects (Ralph-Nearman et al. 2019; Shafran et al. 2008). At the same time, because each body-type category included both male and female body stimuli, the number of same-gender exemplars per body type was limited for any given participant; thus, the present findings should not be interpreted as a direct test of gender-specific appearance-comparison processes. Relatedly, the exploratory analysis including US gender revealed a significant but small four-way interaction for liking ratings, suggesting same-gender body pairings may have been more effective, at least among female participants. However, with only two same-gender exemplars per body-type condition, these cell-level analyses rest on a narrow stimulus base, and replication with a larger and more balanced stimulus set is needed before strong conclusions can be drawn.

The present findings make several theoretical contributions. First, they demonstrate that evaluative conditioning can modify evaluations of neutral stimuli paired with body images, supporting affect-transfer accounts when self-relevance confounds are minimized. Second, they reveal asymmetry in conditioning effectiveness, with positive unconditioned stimuli producing more robust effects than negative stimuli. Third, they identify social comparison tendency as a moderator of overall responsiveness to conditioning procedures, suggesting that person-level factors affecting information processing style may be as important as stimulus-level factors. Fourth, they establish that attribute conditioning effects documented for semantic attributes do not extend to basic perceptual dimensions such as size. Fifth, they advance understanding that individual difference moderators may operate at different levels-social comparison affecting stimulus processing, while self-esteem shows no such influence when stimuli lack self-relevance.

Future research should address several priorities. Testing whether social comparison moderates conditioning with self-relevant conditioned stimuli would determine whether observed moderation generalizes beyond abstract figures. Investigating mechanisms through which social comparison disrupts conditioning-whether through enhanced elaboration, defensive processing, or awareness and reactance-would inform theory and potentially guide interventions. Determining why negative unconditioned stimuli produce weaker conditioning than positive stimuli has implications for conditioning theory broadly. Longitudinal designs assessing persistence beyond immediate post-test are essential before intervention applications. Clinical trials should systematically assess social comparison tendency and explore whether pre-intervention procedures reducing defensive processing enhance conditioning effectiveness, or whether conditioning should target individuals lower in social comparison. Finally, extending dependent measures beyond self-report to include behavioral outcomes would strengthen conclusions about conditioning’s practical significance (Uram et al. 2026).

In conclusion, evaluative conditioning successfully modified evaluations of geometric figures paired with body images, but effects were limited to positive pairings and moderated by social comparison tendency. These findings illuminate boundary conditions of affect transfer while highlighting individual differences’ importance in determining conditioning effectiveness. The laboratory demonstration advances theoretical understanding but leaves open questions about applied utility for body image interventions given constraints imposed by using abstract rather than self-relevant conditioned stimuli.

## Supplementary Information

Below is the link to the electronic supplementary material.

Supplementary Material 1

Supplementary Material

## Data Availability

Data are available upon reasonable request by sending an email to the corresponding author.

## Declarations

### Generative AI in scientific writing

The authors used AI to improve the manuscript’s language and grammar.

## References

[CR1] Aspen V, Martijn C, Alleva JM, Nagel J, Perret C, Purvis C, Saekow J, Lock J, Taylor CB (2015) Decreasing body dissatisfaction using a brief conditioning intervention. Behav Res Ther 69:93–99. 10.1016/j.brat.2015.04.003 25912670

[CR2] Brown Z, Tiggemann M (2016) Attractive celebrity and peer images on Instagram: effect on women’s mood and body image. Body Image 19:37–43. 10.1016/j.bodyim.2016.08.007 27598763

[CR3] F Cash T, Smolak L (2011) Body image: a handbook of science, practise, and prevention (2nd ed). Guilford Press. https://psycnet.apa.org/record/2011-20792-000

[CR4] Davison TE, McCabe MP (2005) Relationships between men’s and women’s body image and their psychological, social, and sexual functioning. Sex Roles 52:463–475. 10.1007/s11199-005-3712-z

[CR6] De Houwer J, Thomas S, Baeyens F (2001) Associative learning of likes and dislikes: a review of 25 years of research on human evaluative conditioning. Psychol Bull 127(6):853–869. 10.1037/0033-2909.127.6.853 11726074

[CR7] de Valle MK, Gallego-García M, Williamson P, Wade TD (2021) Social media, body image, and the question of causation: meta-analyses of experimental and longitudinal evidence. Body Image 39:276–292. 10.1016/j.bodyim.2021.10.001 34695681

[CR8] Dijksterhuis A (2004) I like myself but I don’t know why: enhancing implicit self-esteem by subliminal evaluative conditioning. J Personal Soc Psychol 86(2):345–355. 10.1037/0022-3514.86.2.345 14769089

[CR9] Dwyer DM, Jarratt F, Dick K (2007) Evaluative conditioning with foods as CSs and body shapes as USs: no evidence for sex differences, extinction, or overshadowing. Cogn Emot 21(2):281–299. 10.1080/02699930600551592

[CR10] Fardouly J, Diedrichs PC, Vartanian LR, Halliwell E (2015) Social comparisons on social media: the impact of Facebook on young women’s body image concerns and mood. Body Image 13:38–45. 10.1016/j.bodyim.2014.12.002 25615425

[CR11] Faul F, Erdfelder E, Buchner A, Lang AG (2009) Statistical power analyses using G* Power 3.1: Tests for correlation and regression analyses. Behav Res Methods 41(4):1149–1160. 10.3758/BRM.41.4.1149 19897823

[CR12] Festinger L (1954) A theory of social comparison processes. Hum Relat 7(2):117–140. 10.1177/001872675400700202

[CR13] Fioravanti G, Bocci Benucci S, Ceragioli G, Casale S (2022) How the exposure to beauty ideals on social networking sites influences body image: a systematic review of experimental studies. Adolesc Res Rev 7(3):419–458. 10.1007/s40894-022-00179-4

[CR14] Firestone C, Scholl BJ (2016) Cognition does not affect perception: evaluating the evidence for top-down effects. Behav Brain Sci 39:e229. 10.1017/S0140525X15000965 26189677

[CR15] Fitzsimmons-Craft EE, Bardone-Cone AM, Harney MB (2012) Development and validation of the body, eating, and exercise comparison orientation measure (BEECOM) among college women. Body Image 9(4):476–487. 10.1016/j.bodyim.2012.07.007 22902098

[CR16] Förderer S, Unkelbach C (2015) Attribute conditioning: Changing attribute-assessments through mere pairings. Q J Experimental Psychol 68(1):144–164. 10.1080/17470218.2014.939667 24985786

[CR18] Gawronski B, Walther E (2012) What do memory data tell us about the role of contingency awareness in evaluative conditioning? J Exp Soc Psychol 48(3):617–623. 10.1016/j.jesp.2012.01.002

[CR17] Gawronski B, Balas R, Creighton LA (2014) Can the formation of conditioned attitudes be intentionally controlled? Pers Soc Psychol Bull 40:419–432. 10.1177/0146167213513907 24280393

[CR20] Glashouwer KA, Neimeijer RAM, De Koning ML, Vestjens M, Martijn C (2018) Evaluative conditioning as a body image intervention for adolescents with eating disorders. J Consult Clin Psychol 86(12). 10.1037/ccp0000311 30507229

[CR19] Glashouwer KA, Masselman I, De Jong PJ (2019) Reducing body dissatisfaction by means of an evaluative conditioning procedure in undergraduate women: a replication study. Behav Res Ther 121:103435. 10.1016/j.brat.2019.103435 31488242

[CR21] Halbeisen G, Paslakis G (2021) All i need is two: the clinical potential of adding evaluative pairing procedures to cognitive behavioral therapy for changing self-, body-and food-related evaluations. J Clin Med 10(20):4703. 10.3390/jcm10204703 PMC8539260 34682826

[CR23] Högden F, Stahl C, Unkelbach C (2020) Similarity-based and rule-based generalisation in the acquisition of attitudes via evaluative conditioning. Cogn Emot 34(1):105–127. 10.1080/02699931.2019.1588709 31020918

[CR22] Hofmann W, De Houwer J, Perugini M, Baeyens F, Crombez G (2010) Evaluative conditioning in humans: a meta-analysis. Psychol Bull 136(3):390–421. 10.1037/a0018916 20438144

[CR24] Holland G, Tiggemann M (2016) A systematic review of the impact of the use of social networking sites on body image and disordered eating outcomes. Body Image 17:100–110. 10.1016/j.bodyim.2016.02.008 26995158

[CR5] De Houwer J (2018) Propositional models of evaluative conditioning. Social Psychol Bull 13(3). 10.5964/spb.v13i3.28046

[CR25] Hütter M, Sweldens S (2013) Implicit misattribution of evaluative responses: contingency-unaware evaluative conditioning requires simultaneous stimulus presentations. J Exp Psychol Gen 142(3):638–643. 10.1037/a0029989 22946896

[CR26] Huzoaica FG, De Houwer J, Perugini M, Rusu A, Sava FA (2025) Beyond valence transfer in an evaluative conditioning paradigm: on the nature of the phenomenon and its relation to personality. Collabra: Psychol 11(1). 10.1525/collabra.137108. Scopus

[CR27] Inquisit, Version 6.0 (2023) [OSX]. Millisecond. https://www.millisecond.com

[CR28] Jones CR, Olson MA, Fazio RH (2010) Evaluative conditioning. In: Advances in experimental social psychology, vol. 43. Elsevier. pp. 205–255. 10.1016/S0065-2601(10)43005-1 PMC3254108 22241936

[CR29] Kosinski T (2019) A brief mobile evaluative conditioning app to reduce body dissatisfaction? A pilot study in university women. Front Psychol 10. 10.3389/fpsyg.2019.02594 PMC6881283 31824380

[CR33] Łaguna M, Lachowicz-Tabaczek K, Dzwonkowska I (2007) Skala samooceny SES Morrisa Rosenberga-polska adaptacja metody [SES self-assessment scale by Morris Rosenberg—Polish adaptation of the method]. Psychologia Społeczna 2(2):164–176

[CR31] Lieberman DL, Tybur JM, Latner JD (2012) Disgust sensitivity, obesity stigma, and gender: contamination psychology predicts weight bias for women, not men. Obesity 20(9):1803–1814. 10.1038/oby.2011.247 21836644

[CR32] Lupyan G (2015) Cognitive penetrability of perception in the age of prediction: predictive systems are penetrable systems. Rev Philos Psychol 6(4):547–569. 10.1007/s13164-015-0253-4

[CR34] Martijn C, Vanderlinden M, Roefs A, Huijding J, Jansen A (2010) Increasing body satisfaction of body concerned women through evaluative conditioning using social stimuli. Health Psychol 29(5):514–520. 10.1037/a0020770 20836606

[CR35] Masselman I, de Jong PJ, Glashouwer KA (2022) What’s not to like? Enhancing women’s body satisfaction by means of an evaluative conditioning procedure with positive social feedback. J Experimental Psychopathol 13(1). 10.1177/20438087221085767

[CR36] Mitchell CJ, De Houwer J, Lovibond PF (2009) The propositional nature of human associative learning. Behav Brain Sci 32(2):183–198. 10.1017/S0140525X09000855 19386174

[CR37] Moran T, Nudler Y, Bar-Anan Y (2023) Evaluative conditioning: past, present, and future. Ann Rev Psychol 74:245–269. 10.1146/annurev-psych-032420-031815. Scopus 36130066

[CR38] Myers TA, Crowther JH (2009) Social comparison as a predictor of body dissatisfaction: a meta-analytic review. J Abnorm Psychol 118(4):683–698. 10.1037/a0016763 19899839

[CR39] Pearl RL, Wadden TA, Bach C, Gruber K, Leonard S, Walsh OA, Tronieri JS, Berkowitz RI (2020) Effects of a cognitive-behavioral intervention targeting weight stigma: a randomized controlled trial. J Consult Clin Psychol 88(5):470–480. 10.1037/ccp0000480 PMC7148168 31971410

[CR40] Perloff RM (2014) Social media effects on young women’s body image concerns: theoretical perspectives and an agenda for research. Sex Roles 71(11–12):363–377. 10.1007/s11199-014-0384-6

[CR41] Pylyshyn Z (1999) Is vision continuous with cognition? The case for cognitive impenetrability of visual perception. Behav Brain Sci 22(3):341–365. 10.1017/S0140525X99002022 11301517

[CR42] Quigley M, Dymond S, Kiely K, Bradley A, Haselgrove M (2025) Attribute conditioning is insensitive to cue competition and is not predicted by the big five personality traits. Pers Soc Psychol Bull 01461672241308921. 10.1177/01461672241308921 PMC13022014 39812141

[CR43] Raftopoulos A (2017) Pre-cueing, the epistemic role of early vision, and the cognitive impenetrability of early vision. Front Psychol 8:1156. 10.3389/fpsyg.2017.01156 PMC5502256 28740474

[CR44] Ralph-Nearman C, Achee M, Lapidus R, Stewart JL, Filik R (2019) A systematic and methodological review of attentional biases in eating disorders: food, body, and perfectionism. Brain Behav 9(12):e01458. 10.1002/brb3.1458 PMC6908865 31696674

[CR45] Rochelle TL, Huang X (2025) The influence of gender and body satisfaction on attentional bias among young adults in Hong Kong: an eye-tracking study. J Health Psychol 30(10):2696–2707. 10.1177/13591053241278509 PMC12381397 39277751

[CR63] Rosenberg M (1965) Rosenberg self-esteem scale. J Relig Health. 10.1037/t01038-000

[CR46] Shafran R, Lee M, Cooper Z, Palmer RL, Fairburn CG (2008) Effect of psychological treatment on attentional bias in eating disorders. Int J Eat Disord 41(4):348–354. 10.1002/eat.20500 PMC2798071 18213684

[CR47] Sperlich LM, Unkelbach C (2024) Why is there no negativity bias in evaluative conditioning? A cognitive-ecological answer. J Personal Soc Psychol. 10.1037/pspa0000431 39541524

[CR48] Stice E, Shaw HE (2002) Role of body dissatisfaction in the onset and maintenance of eating pathology. J Psychosom Res 53(5):985–993. 10.1016/S0022-3999(02)00488-9 12445588

[CR49] Thompson JK, Coovert MD, Stormer SM (1999) Body image, social comparison, and eating disturbance: a covariance structure modeling investigation. Int J Eat Disord 26(1):43–51. 10.1002/(SICI)1098-108X(199907)26:1&lt;43::AID-EAT6&gt;3.0.CO;2-R 10349583

[CR50] Tiggemann M, Slater A (2013) NetGirls: The Internet, Facebook, and body image concern in adolescent girls. Int J Eat Disord 46(6):630–633. 10.1002/eat.22141 23712456

[CR52] Unkelbach C, Förderer S (2018) A model of attribute conditioning. Social Psychol Bull 13(3):e28568. 10.5964/spb.v13i3.28568 26865290

[CR51] Unkelbach C, Alves H, Koch A (2020) Negativity bias, positivity bias, and valence asymmetries: Explaining the differential processing of positive and negative information. Adv Exp Soc Psychol 62:115–187. 10.1016/BS.AESP.2020.04.005

[CR53] Uram P, Balas R, Kwiatkowska A, Wąsowicz J, Skalski S (2026) The Polish version of the body, eating, and exercise comparison orientation measure: adaptation, validation, and psychometric evaluation. Health Psychol Rep. 10.5114/hpr/214692

[CR64] Uram P, Skalski-Bednarz SB, Sava F, Balas R (2026) Evaluative conditioning of attitudes towards nutrition: a systematic review of research using food and body stimuli. Pol Psychol Bull 57:71–87. 10.24425/ppb.2026.153994

[CR54] Vartanian LR (2010) Disgust and perceived control in attitudes toward obese people. Int J Obes 34(8):1302–1307. 10.1038/ijo.2010.45 20195287

[CR56] Vartanian LR, Dey S (2013) Self-concept clarity, thin-ideal internalization, and appearance-related social comparison as predictors of body dissatisfaction. Body Image 10(4):495–500. 10.1016/j.bodyim.2013.05.004 23809858

[CR57] Vogel EA, Rose JP, Roberts LR, Eckles K (2014) Social comparison, social media, and self-esteem. Psychol Popular Media Cult 3(4):206–222. 10.1037/ppm0000047

[CR58] Walsh K, McGovern DP, Dully J, Kelly SP, O’Connell RG (2024) Prior probability cues bias sensory encoding with increasing task exposure. eLife 12:RP91135. 10.7554/eLife.91135.3 PMC10987094 38564237

[CR59] Walther E, Gawronski B, Blank H, Langer T (2009) Changing likes and dislikes through the back door: the US-revaluation effect. Cogn Emot 23(5):889–917. 10.1080/02699930802212423

[CR60] West BT, Ghimire D, Axinn WG (2015) Evaluating a modular design approach to collecting survey data using text messages. Surv Res Methods 9(2):111–123. 10.18148/srm/2015.v9i2.6135 PMC4551499 26322137

[CR61] Wilhelm L, Hartmann AS, Becker JC, Vocks S (2019) Thin media images decrease women’s body satisfaction: comparisons between veiled Muslim women, Christian women and atheist women regarding trait and state body image. Front Psychol 10:1074. 10.3389/fpsyg.2019.01074 PMC6524407 31133951

[CR62] Zajonc RB (2001) Mere exposure: a gateway to the subliminal. Curr Dir Psychol Sci 10(6):224–228. 10.1111/1467-8721.00154

