# Supplementary material for: Shaping attitudes toward body-related cues via evaluative conditioning: investigating the roles of social comparison and self-esteem
Source: Cogn Process. 2026 Jul 11;27(4):1235–50. doi: 10.1007/s10339-026-01376-y (PMC13714012; doi:10.1007/s10339-026-01376-y)
Supplement: Supplementary file 1 [file 10339_2026_1376_MOESM1_ESM.docx]

Supplementary Materials

**Stimuli Characteristics**

The conditioned stimuli (CS) were eight neutral geometric figures taken from Gawronski, Balas and Creighton (2014) , presented as individual PNG image files within the task software. The unconditioned stimuli (US) were eight photographs of human bodies varying in weight status and selected to resemble as closely as possible the body stimuli described by Dwyer et al. (2007). Four depicted average-weight bodies (two male, two female), and four depicted obese-weight bodies (two male, two female). All body images were selected to resemble as closely as possible the stimuli described by Dwyer et al. (2007), particularly with respect to pose (standing, front-facing), relatively limited clothing coverage, and background. In addition, the images were framed so that the faces were not visible. All stimuli were presented centrally against a white background, and the display background used during stimulus presentation was also white. The source images were prepared to the same nominal dimensions (50 × 50 mm). In addition, the stimuli were made as comparable as possible with respect to nominal display parameters, cropping, the proportion of screen space occupied by the image, and height-to-width ratio.

Supplementary Results

**Association between valence and liking**

To assess the intra-individual relationship between the two primary rating types, a repeated-measures correlation was conducted across all baseline CS ratings. The analysis revealed a moderate, positive association between valence and liking ratings, rrm(984) = .31, 95% CI [.25, .36], p < .001. This indicates that for any given participant, stimuli rated more positively in valence also tended to be rated as more liked.

**Manipulation check**

To validate the experimental manipulation, we analyzed participants’ initial ratings of the unconditioned stimuli (US). We conducted two 2 (Body Type: Average, Obese) × 2 (Stimulus Gender: Male, Female) × 2 (Participant Gender: Male, Female) mixed ANOVAs, with Body Type and Stimulus Gender as within-subjects factors and Participant Gender as a between-subjects factor. Separate ANOVAs were run for the dependent variables of valence and liking ratings.

The analysis of valence ratings revealed a significant three-way interaction between Body Type, Stimulus Gender, and Participant Gender, F(1, 141) = 9.70, p = .002, ges = .006 (Figure 1). This interaction was qualified by significant main effects of Body Type, F(1, 141) = 334.30, p < .001, ges = .51, and Stimulus Gender, F(1, 141) = 26.86, p < .001, ges = .02, as well as a significant two-way interaction between Body Type and Stimulus Gender, F(1, 141) = 5.74, p = .018, ges = .004. There was also a main effect of Participant Gender, F(1, 141) = 4.04, p = .046, ges = .01.

Figure 1. Interaction of Body Type, Stimulus Gender, and Participant Gender on Pre-Conditioning Valence Ratings. *Note: Mean pre-conditioning valence ratings for average- and obese-body stimuli, separated by stimulus gender. The left panel displays ratings from female participants, and the right panel displays ratings from male participants. Error bars represent ±1 standard error of the mean (SEM).*

To decompose the three-way interaction, we examined the pattern of ratings for male and female participants separately. The core effect of Body Type was exceptionally strong and consistent: across all conditions, average-weight bodies (M = 4.22, SD = 3.12) were rated far more positively than obese bodies (M = - 4.08, SD = 4.11), t(142) = 18.40, p < .001. Bonferroni-corrected one-sample t-tests confirmed that all four US categories were rated significantly different from the neutral point of 0 (ps < .001). Specifically, both average-female and average-male bodies were rated positively, while both obese-female and obese-male bodies were rated negatively, confirming the intended valence of the stimuli.

A similar pattern emerged for liking ratings. The analysis revealed a significant three-way interaction between Body Type, Stimulus Gender, and Participant Gender, F(1, 141) = 10.55, p = .001, ges = .006 (see Figure 2). This was qualified by significant main effects of Body Type, F(1, 141) = 351.92, p < .001, ges = .52, and Stimulus Gender, F(1, 141) = 26.64, p < .001, ges = .02. There were also significant two-way interactions between Body Type and Participant Gender, F(1, 141) = 4.40, p = .038, ges = .01, and between Body Type and Stimulus Gender, F(1, 141) = 8.72, p = .004, ges = .005.

Figure 2. Interaction of Body Type, Stimulus Gender, and Participant Gender on Pre-Conditioning Liking Ratings. *Note: Mean pre-conditioning liking ratings for average- and obese-body stimuli, separated by stimulus gender. The left panel displays ratings from female participants, and the right panel displays ratings from male participants. Error bars represent ±1 standard error of the mean (SEM).*

As with valence, the main effect of Body Type was robust: average-weight bodies (M = 4.01, SD = 3.24) were liked significantly more than obese bodies (M = -4.37, SD = 3.95), t(142) = 18.70, p < .001. Bonferroni-corrected one-sample t-tests confirmed that ratings for all four stimulus categories differed significantly from the neutral point of 0 (ps < .001) in the expected directions.

**Baseline CS Ratings**

To verify that the eight conditioned stimuli (CS) were neutral and equivalent before conditioning, we analyzed baseline valence and liking ratings. We conducted two separate analyses, one for valence and one for liking. First, to test for equivalence among the eight CS items, we fit a linear mixed-effects model for each rating type, with the CS item as a fixed effect and a random intercept for participants. An ANOVA on this model provided an omnibus test for differences among the stimuli. Second, to test for neutrality, we conducted a series of one-sample t-tests, comparing the mean rating of each CS against the neutral point of 0. The resulting p-values were adjusted using the Holm-Bonferroni method to control for multiple comparisons.

For valence ratings, the omnibus ANOVA revealed small but statistically significant differences among the stimuli, F(7, 991.62) = 2.63, p = .011. However, the follow-up one-sample t-tests, corrected for multiple comparisons, showed that none of the eight CSs were rated significantly different from the neutral point of 0 (all ps_adj_ > .12). For liking ratings, the omnibus ANOVA was not significant, F(7, 997.82) = 1.34, p = .228, indicating that the stimuli were rated as equivalently likable. The subsequent Holm-corrected one-sample t-tests confirmed that none of the CSs differed significantly from neutral in terms of liking (all ps_adj_ > .99). Taken together, these results support the assumption that all CSs were functionally neutral at baseline (see Figure 3).

Figure 3. Mean Baseline Valence and Liking Ratings for Each Conditioned Stimulus (CS). *Note: The bars represent the mean pre-conditioning rating for each CS item on a scale from -10 to +10, where 0 represents neutrality. Error bars represent 95% confidence intervals.*

**Size Perception Analyses**

To further probe the null finding from the primary ANOVA, we conducted a series of additional analyses on the change in size perception from pre- to post-conditioning. First, a direct paired-samples t-test comparing the change scores for CSs paired with average versus obese bodies confirmed the ANOVA result: the interaction effect was not significant for either perceived width, t(145) = -1.27, p = .206, or perceived height, t(145) = 0.84, p = .401.

Second, we conducted formal equivalence tests (TOST) to determine if we could reject the presence of a practically meaningful effect, which we defined as a differential change greater than ±5 mm. For perceived width, the equivalence test was non-significant (p = .147), meaning we cannot conclude the effect is statistically equivalent to zero. For perceived height, however, the equivalence test was significant (p = .018), allowing us to reject the presence of an effect larger than ±5 mm. This provides evidence that any effect of conditioning on perceived height was practically meaningless.

Finally, a Bayesian analysis was conducted to quantify the evidence for the null hypothesis (i.e., no difference between the change scores) over the alternative hypothesis. For perceived width, the analysis yielded a Bayes Factor in favor of the null hypothesis of BF₀₁ = 4.93, indicating that the data are approximately 5 times more likely under the null hypothesis of no effect than under the alternative hypothesis. For perceived height, the evidence for the null was even stronger, with a BF₀₁ = 7.66. According to established conventions, these Bayes Factors provide moderate evidence for the null hypothesis. Taken together, these converging frequentist and Bayesian analyses provide strong support for the conclusion that the evaluative conditioning manipulation did not influence participants' perception of stimulus size.

**Participant Sex and Individual-Difference Moderation**

We examined the exploratory individual-difference analyses with participant sex included. Because social comparison (BEECOM) and self-esteem (SES) are person-level variables, the moderator × sex question was tested at the participant level. For each participant we computed the pre-to-post change in CS evaluation, summarised as (a) *overall* responsiveness, the mean change across the average-paired and obese-paired conditions, and (b) a *differential* conditioning score, the average-paired change minus the obese-paired change (equivalent to the Time × Paired Condition contrast). Each change score was regressed on the centred moderator, effect-coded sex, and their interaction. Equivalent item-level linear mixed models with sex added to the Time × Paired Condition × moderator structure (random intercepts for participant and CS) yielded the same conclusions and are provided in the analysis script. Participants who selected “prefer not to say” (*n* = 3) were excluded from these analyses (*n* = 143). Given the number of tests, we applied a Holm correction across the family of eight moderator × sex interactions.

Moderator × Sex interactions

Table 1 reports the eight interaction tests. Only one was significant: the BEECOM × Sex interaction on *overall* liking responsiveness, *b* = 3.04, *t*(139) = 3.11, *p* = .002 (Holm-adjusted *p* = .018). No interaction involving self-esteem was significant, the BEECOM × Sex interaction for valence was not significant, and sex did not qualify the *differential* conditioning effect for either outcome.

Table 1. Participant Sex as a Moderator of Individual-Difference Effects on Conditioning-Related Change

| **Outcome / effect** | **Moderator × Sex** | **b** | **t(139)** | **p** |
| --- | --- | --- | --- | --- |
| Liking - overall responsiveness | BEECOM × Sex | +3.04 | 3.11 | .002 |
| Liking - overall responsiveness | SES × Sex | −0.34 | −0.34 | .735 |
| Liking - differential conditioning | BEECOM × Sex | −0.27 | −0.20 | .839 |
| Liking - differential conditioning | SES × Sex | +0.77 | 0.59 | .555 |
| Valence - overall responsiveness | BEECOM × Sex | +1.57 | 1.59 | .113 |
| Valence - overall responsiveness | SES × Sex | +0.42 | 0.43 | .668 |
| Valence - differential conditioning | BEECOM × Sex | +0.36 | 0.34 | .736 |
| Valence - differential conditioning | SES × Sex | +0.91 | 0.87 | .388 |

*Note. b = unstandardised regression coefficient for the interaction; effect-coded sex (female = −0.5, male = +0.5). Overall responsiveness = mean pre-to-post change across conditions; differential conditioning = average-paired minus obese-paired change.*

**Simple slopes for the significant interaction**

Decomposing the BEECOM × Sex interaction on overall liking change, higher social comparison was associated with *smaller* increases in liking among women (simple slope = −2.09, *r* = −.28, *p* = .007; mean change = +0.44), replicating the overall moderation reported in the main text. Among men the association was non-significant and opposite in sign (simple slope = 0.96, *r* = .25, *p* = .074; mean change = −0.07).

**Sex main effects on the conditioning effect**

For completeness, participant sex did not have a main effect on the conditioning effect: neither overall responsiveness nor the differential score differed by sex for liking (overall, *t*(141) = −1.00, *p* = .320; differential, *t*(141) = 0.44, *p* = .662) or valence (overall, *t*(141) = 1.13, *p* = .262; differential, *t*(141) = 0.63, *p* = .531).

**Interpretation**

Including participant sex does not change the manuscript’s conclusions. Social comparison continues to moderate *general* responsiveness to the procedure rather than the differential conditioning effect, and self-esteem remains a non-moderator. The one new effect - that the social-comparison moderation of overall liking change is carried by women - is consistent with same-sex appearance-comparison accounts, but is based on an unbalanced and modest male subsample (*n* = 52) and a non-significant, oppositely signed male trend. We therefore regard it as an exploratory finding warranting confirmatory, sex-balanced replication.

**Effect sizes for baseline CS equivalence tests**

Table 2 reports partial η² (with 90% confidence intervals) for the baseline omnibus tests of CS equivalence, computed from the linear mixed models (CS item as a fixed effect, random intercept for participant). All significant baseline differences are small in magnitude; perceived height shows the largest difference (small-to-medium).

Table 2. Partial Eta-Squared Effect Sizes for Baseline CS Equivalence Tests

| **Baseline dimension** | **Omnibus test** | **η²ₚ** | **90% CI** |
| --- | --- | --- | --- |
| Valence | F(7, 991.62) = 2.63, p = .011 | .018 | [.002, .027] |
| Liking | F(7, 997.82) = 1.34, p = .228 | .009 | [.000, .014] |
| Perceived width | F(7, 1015) = 3.54, p = .001 | .024 | [.006, .035] |
| Perceived height | F(7, 1015) = 8.45, p < .001 | .055 | [.029, .073] |

*Note. η²ₚ = partial eta squared, from effect size:F_to_eta2 using the model F and degrees of freedom. Benchmarks: .01 small, .06 medium, .14 large.*
